# Myristic acid potentiates palmitic acid-induced lipotoxicity and steatohepatitis associated with lipodystrophy by sustaning de novo ceramide synthesis

**DOI:** 10.18632/oncotarget.6286

**Published:** 2015-11-02

**Authors:** Laura Martínez, Sandra Torres, Anna Baulies, Cristina Alarcón-Vila, Montserrat Elena, Gemma Fabriàs, Josefina Casas, Joan Caballeria, Jose C. Fernandez-Checa, Carmen García-Ruiz

**Affiliations:** ^1^ Cell Death and Proliferation, Institute of Biomedical Research of Barcelona (IIBB), CSIC, Barcelona, Spain; ^2^ Liver Unit, Hospital Clinic I Provincial de Barcelona, IDIBAPS and CIBERehd, Barcelona, Spain; ^3^ Biomedic Diagnosis Center, Hospital Clinic i Provincial de Barcelona, IDIBAPS, Barcelona, Spain; ^4^ Research Unit on BioActive Molecules (RUBAM), Departament de Química Orgànica Biològica, Institut d'Investigacions Químiques i Ambientals de Barcelona, Consejo Superior de Investigaciones Científicas (CSIC), Barcelona, Spain; ^5^ Research Center for ALPD, Keck School of Medicine, Univerisity of Southern California, Los Angeles, CA, USA

**Keywords:** sphingolipid, hepatocyte, FFA, endoplasmic reticulum, NAFLD, Pathology Section

## Abstract

Palmitic acid (PA) induces hepatocyte apoptosis and fuels de novo ceramide synthesis in the endoplasmic reticulum (ER). Myristic acid (MA), a free fatty acid highly abundant in copra/palmist oils, is a predictor of nonalcoholic steatohepatitis (NASH) and stimulates ceramide synthesis. Here we investigated the synergism between MA and PA in ceramide synthesis, ER stress, lipotoxicity and NASH. Unlike PA, MA is not lipotoxic but potentiated PA-mediated lipoapoptosis, ER stress, caspase-3 activation and cytochrome c release in primary mouse hepatocytes (PMH). Moreover, MA kinetically sustained PA-induced total ceramide content by stimulating dehydroceramide desaturase and switched the ceramide profile from decreased to increased ceramide 14:0/ceramide16:0, without changing medium and long-chain ceramide species. PMH were more sensitive to equimolar ceramide14:0/ceramide16:0 exposure, which mimics the outcome of PA plus MA treatment on ceramide homeostasis, than to either ceramide alone. Treatment with myriocin to inhibit ceramide synthesis and tauroursodeoxycholic acid to prevent ER stress ameliorated PA plus MA induced apoptosis, similar to the protection afforded by the antioxidant BHA, the pan-caspase inhibitor z-VAD-Fmk and JNK inhibition. Moreover, ruthenium red protected PMH against PA and MA-induced cell death. Recapitulating *in vitro* findings, mice fed a diet enriched in PA plus MA exhibited lipodystrophy, hepatosplenomegaly, increased liver ceramide content and cholesterol levels, ER stress, liver damage, inflammation and fibrosis compared to mice fed diets enriched in PA or MA alone. The deleterious effects of PA plus MA-enriched diet were largely prevented by *in vivo* myriocin treatment. These findings indicate a causal link between ceramide synthesis and ER stress in lipotoxicity, and imply that the consumption of diets enriched in MA and PA can cause NASH associated with lipodystrophy.

## INTRODUCTION

Nonalcoholic steatohepatitis (NASH) is an advanced stage of fatty liver disease that can progress to cirrhosis and liver cancer. NASH is associated with obesity, lipodystrophy and type-2 diabetes. Hepatocyte apoptosis is a multifaceted event of relevance for NASH [1]. Palmitic acid (PA) is the most abundant saturated fatty acid (SFA) found in Western diets and processed foods and it is known to cause lipoapoptosis in hepatocytes through different mechanisms, including endoplasmic reticulum (ER) stress, mitochondrial dysfunction, JNK activation, lysosomal membrane permeabilization and death receptor 5 (DR5) activation [2–9]. Palmitoyl-CoA derived from PA is a precursor of ceramide species, a heterogeneous family of sphingolipids present in biological membranes that define specific microdomains involved in signaling cascades [10–12]. De novo ceramide synthesis takes place in the ER and begins with the condensation of the amino acid L-serine with palmitoyl-CoA to form 3-ketosphinganine, in a reaction catalyzed by the rate-limiting enzyme serine palmitoyltransferase (SPT). Reduction of 3-ketosphinganine to sphinganine followed by acylation with fatty acyl chains of varying lengths by ceramide synthases (CerS) generates dihydroceramide [13, 14]. Finally, dihydroceramide desaturase (DES) creates a double trans4-5 bond thereby converting dihydroceramide into ceramide. Besides its function in membrane biophysics and function, ceramide has been recognized as a second messenger involved in multiple cell processes, including cell stress and cell death, senescence and differentiation, ER stress, autophagy and disruption of mitochondrial function [10, 12, 15–17].

Myristic acid (MA) is a SFA found in small amounts (< 1% of total free fatty acids, FFAs) in animal tissues. However, MA is highly abundant in milk fat (7-12%) and, especially, in copra and palmist oils where MA can reach up to 23% of total fatty acids [18]. MA has recently been identified as a predictor of NASH, as increased serum MA levels have been reported in patients with NASH compared to subjects with simple steatosis and correlated with fibrosis stage [18]. Dietary MA has been shown to accumulate in liver and adipose tissue in rats [19]. Moreover, *in situ* lipidomic analysis by cluster TOF-SIMS imaging revealed the presence of MA and other SFA in steatotic areas in patients with fatty livers [20]. Furthermore, previous studies reported that the N-terminal myristoylation of DES increased DES activity in COS-7 cells, thereby stimulating ceramide synthesis [21]. Since the contribution of ceramide to ER stress caused by PA has been poorly characterized and because the interplay between PA and MA in hepatocyte lipoapoptosis has not been previously examined, the purpose of our study was to explore whether MA synergizes with PA to cause lipoapoptosis in hepatocytes. Moreover, mice were fed a diet enriched in both MA and PA to examine the course of NASH progression. Our findings show that MA potentiates PA-induced lipoapoptosis in primary mouse hepatocytes (PMH) and mice fed a diet enriched in MA and PA exhibits lipodystrophy, increased hepatic ceramide and cholesterol levels, liver injury, inflammation and fibrosis, effects that were prevented by inhibition of the novo ceramide synthesis. These results indicate that the consumption of diets enriched in MA (copra/palmist oils) and PA (western/processed foods) may lead to NASH associated with lipodystrophy.

## RESULTS

### PA plus MA cause mild steatosis and generate selective ceramide species

**Figure 1:**
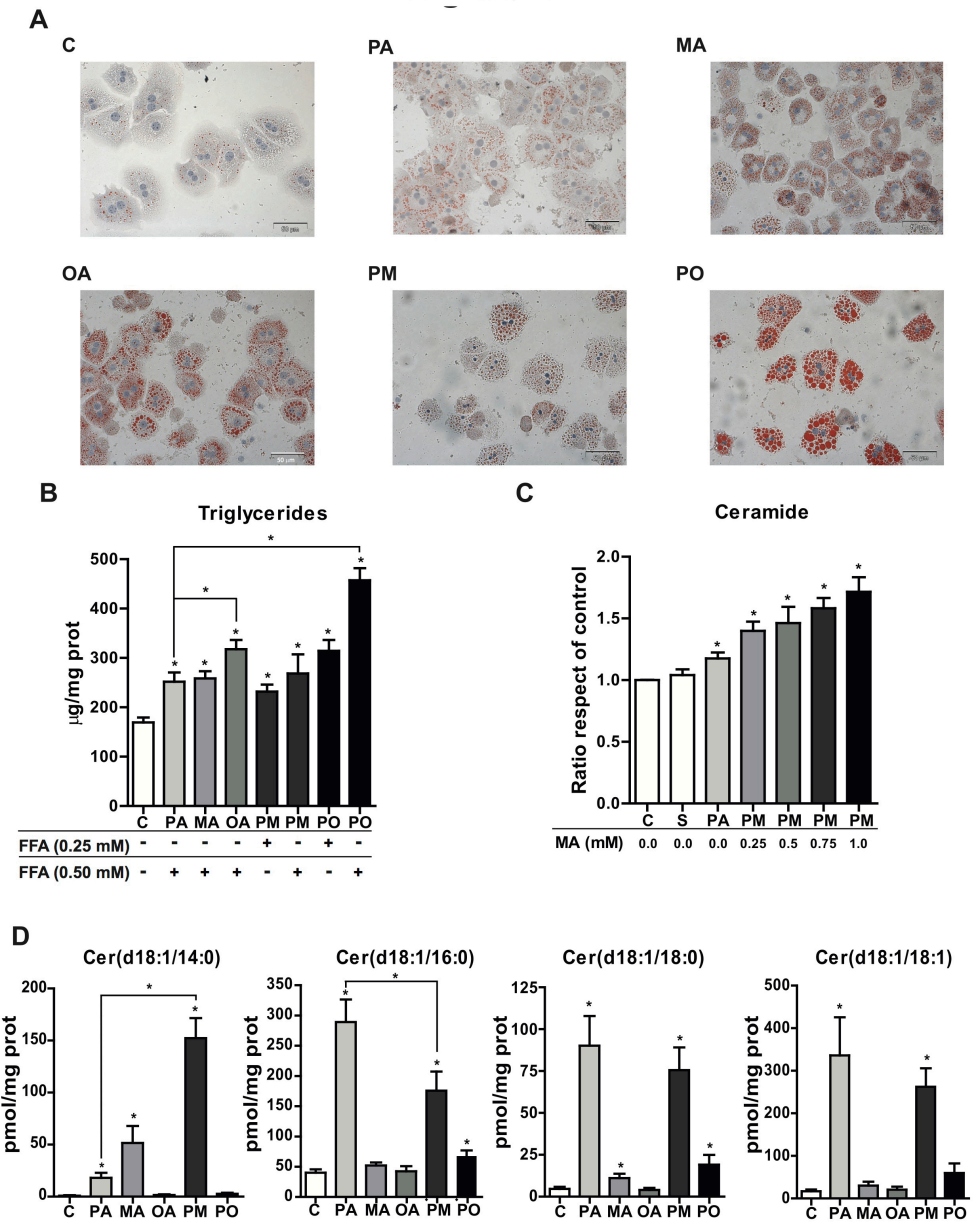
Effect of PA and MA in TG levels and ceramide species PMH were incubated with PA, MA or OA and the combination PA plus MA (PM) and PA plus OA (PO) at 0.5mM overnigh. PMH were processed for oil-red staining **A.** and for the determination of TG levels at the concentrations indicated **B.** In some cases, PMH were treated with 0.5mM PA and with increasing MA doses as indicated to determine total ceramide levels **C.** Moreover, lipid extracts were analyzed by mass spectrometry to determine the relative abundance of different ceramide species **D.** Results are the mean±SEM of *N* = 5-6 individual experiments. *p < 0.05 *vs*. control and PA-treated PMH.

MA is little abundant in mammalian tissues but highly enriched in milk fat and, particularly, in copra and palmist oils where MA accounts for up to 23% of total free fatty acids. To examine the impact of MA in modulating the lipotoxic effect of PA, we used MA at mM concentration to mimic the exposure resulting from the consumption of diets enriched in MA (e.g. copra/palmist oils); moreover, to investigate the effect of MA-rich milk fat-based diet in diabetic cardiomyopathy, Russo et al incubated cardiomyocytes with 1.5 mM MA [22]. We first compared the effect of PA, MA and their combination PA plus MA (PM) in intracellular lipid content in PMH with respect to that caused by the unsaturated fatty acid oleic acid (OA). Control treatment (BSA alone) showed scarce lipid accumulation in the cytoplasm of PMH as evidenced by oil-red staining, whereas treatments with PA or MA caused mild steatosis compared to that elicited by OA (Figure 1A). Combination of PA with OA (PO) resulted in increased macrovesicular steatosis with respect to either PA or OA alone, which contrasts with the onset induced by the combination of PA plus MA (Figure 1A). Determination of triglyceride (TG) levels indicated that OA and its combination with PA (PO) promoted more TG accumulation than either fatty acid alone (Figure 1B). The effect of MA plus OA in oil-red staining and TG levels was similar to that caused by PA and OA (not shown).

We next analyzed ceramide levels following FFAs exposure. Kinetic analyses of ceramide generation indicated that in contrast to the accumulation of TG, OA did not increase total ceramide content compared to PA or MA alone and especially their combination PA plus MA (PM), whose effect in increasing total ceramide mass increased over time and peaked after 12 hour of incubation (Supplemental Figure 1), indicating the ability of PA plus MA to kinetically sustain ceramide generation. In order to further analyze the involvement of MA in ceramide production, cells were treated with a constant dose of PA and increasing doses of MA, and total ceramide levels were quantified by HPLC. As seen, total ceramide levels augmented with increasing MA concentrations, indicating the potentiation of PA-induced ceramide synthesis by MA (Figure 1C).

To characterize the individual ceramide species generated in response to PA plus MA in comparison with PA, samples were analyzed by mass spectrometry. The content of long-chain ceramides (C20-C25) (Supplemental Figure 2) as well as medium-chain ceramides (18:0 and 18:1) (Figure 1D) induced by PA with or without MA did not differ significantly, and similar changes were observed in glucosylceramides (Supplemental Figure 3). As for shorter chain ceramides, PA alone caused a predominant increase in ceramide 16:0, which significantly decreased by the combination of PA and MA (Figure 1D). Moreover, MA alone induced a significant increase in ceramide 14:0 compared to PA, which further increased (3-4 fold) in the presence of PA (PM) (Figure 1D). Thus, the combination PA and MA generated ceramide 14:0 and ceramide 16:0 at equimolar concentration. As expected, treatment of hepatocytes with OA barely changed ceramide levels. These findings indicate that PA plus MA modestly increase TG but enhance ceramide levels whose profile switches from decreased ceramide 14:0/16:0 by PA alone to increased ceramide 14:0/16:0 by PA plus MA.

### MA exacerbates PA-induced lipoapoptosis

**Figure 2:**
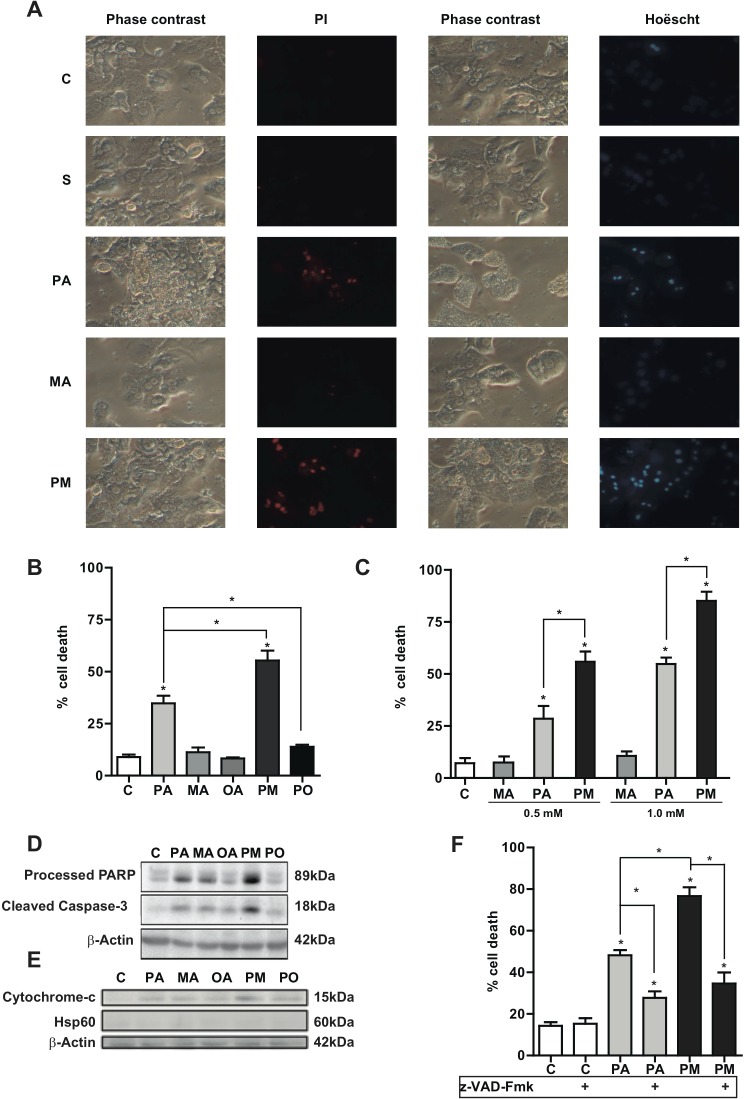
Effect of PA and MA in hepatocyte lipoapoptosis PMH were treated with PA, MA and combination PA plus MA (PM) and cells were examine by phase contrast and staining with propidium iodide (PI) or Hoëscht **A.** Alternatively, cell death caused by PA and MA was determined by trypan blue exclusion to determine percentage of cell death **B.** and **C.** In some cases, extracts were processed for the determination of PARP processing and caspase 3 cleavage **D.**, as well as cytochrome c release **E.** The effect of pan-caspase inhibitor z-VAD-Fmk (15μM) on PA and PA plus MA (PM) was determined **F.** The Results are the mean±SEM of *N* = 6-8 individual experiments. *p < 0.05 *vs*. control and PA-treated PMH.

PA is a lipotoxic SFA that targets different organelles, including ER and mitochondria mediating hepatocyte apoptosis [6, 7]. To address the synergism between PA and MA in lipoapoptosis, we used equimolar concentrations of both fatty acids at a relevant concentration found in western diets (PA) and copra/palmist oils (MA). Unlike PA, MA alone did not kill PMH as indicated by propidium iodide and Hoescht staining (Figure 2A). However, the combination of PA plus MA significantly increased cell death compared to PA alone (Figure 2B). As seen, the lipotoxicity of PA augmented with increasing doses of MA (Figure 2C). Moreover, while MA did not kill hepatocytes at any concentration, 0.5mM PA in the presence of MA (0.5mM) (black column Figure 2C) killed more hepatocytes than 0.5mM PA alone and similar to the degree of cell death caused by 1.0mM PA alone (light grey column Figure 2C), indicating a synergistic effect of MA in PA-induced lipotoxicity. Moreover, cell death determined by the extracellular release of glutathione-S-transferase (GST), as described in Supplemental Methods, showed similar results as those observed by propidium iodide/Hoescht staining. Further, caspase 3 activity determined by release of 7-amino-4-trifluoromethyl coumarin from Ac-DEVD-AMC (not shown) confirmed the potentiation of PA-induced lipoapoptosis by MA. Consistent with the role of OA in increasing TG levels but not ceramide, co-incubation of PA with OA (PO) prevented cell death by PA (Figure 2B). Cerulenin, a specific fatty acid synthase inhibitor [23], did not affect the lipotoxicity of PA plus MA (not shown), suggesting that the potentiation of PA-mediated cell death by MA was not due to newly synthesized PA.

To further characterize the type of cell death caused by PA and MA we analyzed signs of apoptotic cell death, including the status of caspase-3 cleavage and cytochrome c release. As seen, PA plus MA (PM) increased the level of caspase 3 cleavage as well as the processing of PARP, a target of active caspase 3, with respect to PA alone (Figure 2D). Consistent with these findings, PA plus MA enhanced the release of cytochrome c from mitochondria to the cytosol (Figure 2E) compared to PA alone. As expected from these findings the pan-caspase inhibitor z-VAD-Fmk significantly ameliorated PA plus MA-induced hepatocellular apoptosis (Figure 2F). In line with the switched ceramide profile caused by PA plus MA with respect to PA alone, equimolar ceramide 14:0 and ceramide 16:0, mimicking the combination of PA with MA, caused more cell death in PMH than either ceramide species separately (Supplemental Figure 4A). In addition, consistent with previous findings [21], MA but not PA dose-dependently stimulated DES activity (Supplemental Figure 4B), in line with the kinetically sustained generation of total ceramide levels (Supplemental Figure 1). These findings underscore that MA potentiates PA-induced hepatocyte apoptosis and involves the mitochondrial apoptotic pathway.

### MA potentiates PA-induced reactive oxygen species through *de novo* ceramide synthesis

**Figure 3:**
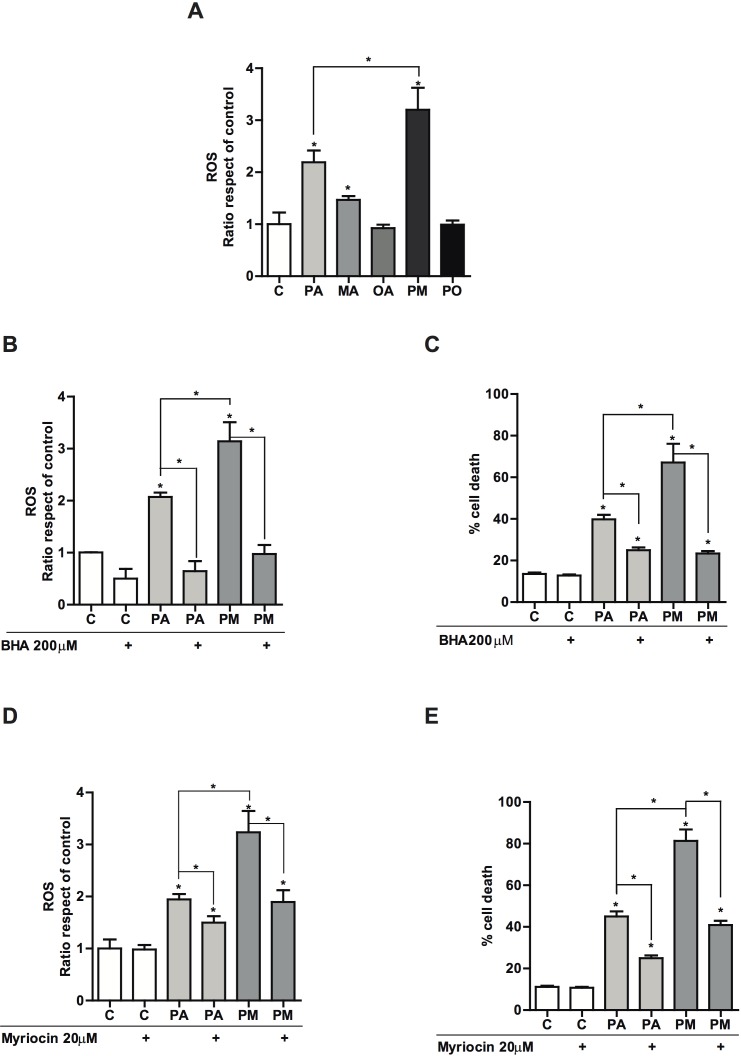
Effect of the antioxidant BHA and the SPT inhibitor myriocin in PA and PA plus MA-induced reactive oxygen species and cell death PMH were treated with PA with or without MA (0.5mM each) for 12 hours. Cell death was then evaluated by trypan blue exclusion **A.** In some cases, cells were incubated with BHA (100μΜ) in the presence of PA and PA plus MA and ROS generation by DCF **B.** and cell death **C.** were determined. Alternatively, PMH were treated with myriocin (20μΜ) to block de novo ceramide synthesis and its effect on ROS generation **D.** and cell death **E.** were analyzed. Results are the mean±SEM of *N* = 5-6 individual experiments. *p < 0.05 *vs*. control and PA-treated PMH.

Since PA plus MA targeted the mitochondrial apoptosis pathway, we next addressed whether the combination of these fatty acids induced the generation of reactive oxygen species (ROS). As shown, treatment with PA alone increased ROS generation determined by dichlorofluorescein-diacetate, and this effect was enhanced by the combination of PA plus MA (PM) (Figure 3A). The presence of the unsaturated fatty acid OA prevented PA-induced ROS production (Figure 3A). To examine the impact of ROS generation in PA plus MA induced lipotoxicity, we examined the effect of the antioxidant BHA. The incubation with BHA suppressed PA and PA plus MA-mediated ROS overproduction (Figure 3B) and protected PMH against PA and PA plus MA-induced cell death (Figure 3C). To determine whether ceramide synthesis contributed to the oxidative stress caused by PA with or without MA, PMH were pretreated with myriocin, a specific SPT inhibitor, which prevents *de novo* ceramide synthesis [24]. In line with previous findings [24], myriocin inhibited SPT activity by 90% based on the conversion of water-soluble [3H]serine to the chloroform-soluble product, 3-ketodihydrosphingosine. Importantly, myriocin significantly reduced ROS generation and protected against PA plus MA-induced cell death in response to PA and PA plus MA (Figure 3D, 3E). Silencing SPT by siRNA reproduced the protective effects of myriocin on ROS generation and cell death caused by PA plus MA (not shown). These findings indicate that oxidative stress caused by *de novo* ceramide synthesis contributes to cell death induced by PA plus MA.

### MA enhances PA-induced ER stress

**Figure 4:**
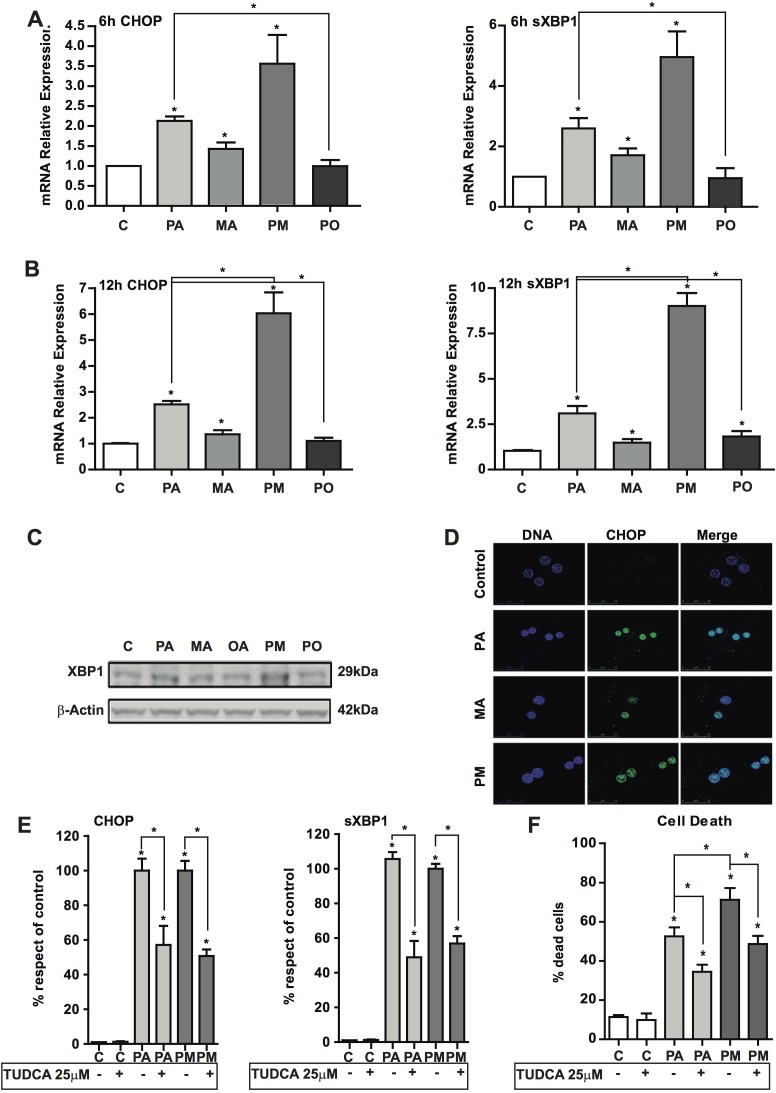
ER stress induction by PA and PA plus MA and the effect of TUDCA PMH were treated with PA and PA plus MA (0.5mM) to determine the expression of ER stress markers at 6 hr **A.** and 12 hr **B.** post treatment. sXBP-1 levels from PMH after incubation with PA and PA plus MA (PM) for 12 hours **C.** Confocal imaging of CHOP and nuclei of PMH treated with PA and PA plus MA (PM). Nuclei were visualized after staining with Hoëscht. In some cases, PMH were treated in the absence or presence of TUDCA (25μM) to determine its impact on CHOP and sXBP-1 expression **E.** as well as cell death **F.** Results are the mean±SEM of *N* = 5-7 individual experiments. *p < 0.05 *vs*. control and PA-treated PMH.

**Figure 5:**
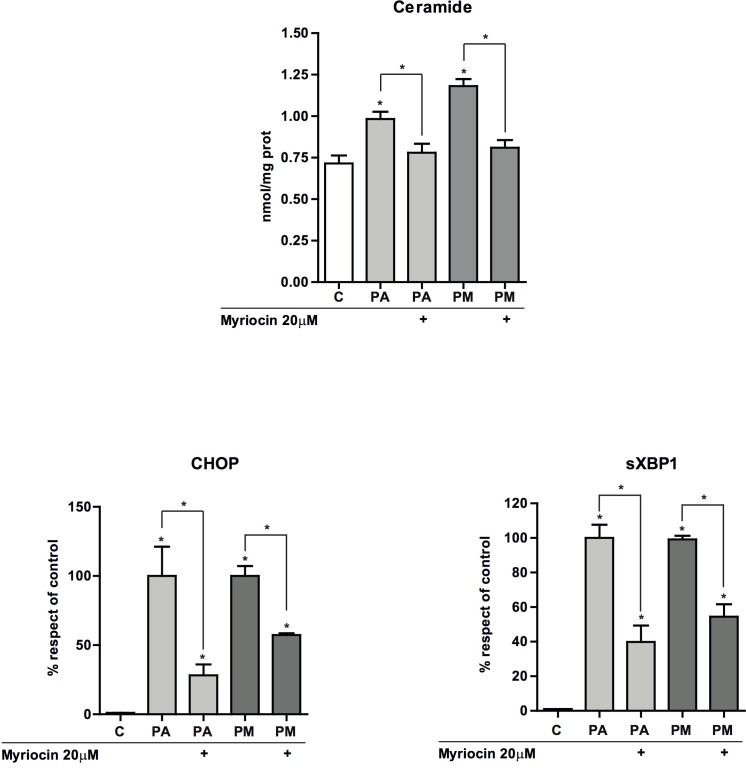
Effect of myriocin on total hepatic ceramide levels and ER stress PMH were incubated with PA with or without MA (0.5mM) each for 6 hours in the presence of myriocin (20μM) and processed for total ceramide determination by HPLC **A.** Alternatively, expression of CHOP **B.** and sXBP-1 **C.** was examined by RT-PCR with or without myriocin. Results are the mean±SEM of *N* = 5-7 individual experiments. *p < 0.05 *vs*. control and PA-treated PMH.

ER stress has been involved in the pathogenesis of NASH [25, 26]. In addition to its role in restoring homeostasis, persistent ER stress induction triggers apoptosis. Therefore, we monitored ER stress markers in PMH after incubation with PA with or without MA. Exposure of hepatocytes to PA with or without MA for 1-2 hours failed to induce ER stress markers, consistent with the lack of early ceramide generation (not shown). However, after 6 hours of incubation, PA induced the expression of ATF4 (not shown), CHOP and sXBP-1 at the mRNA level (Figure 4A, 4B). In line with the mild effect in total ceramide levels, MA alone caused a small increase in the expression of ER stress markers (Figure 4A, 4B) that was not enough to cause cell death (Figure 2B, 2C), suggesting a threshold between ceramide generation, the induction of ER stress and cell death. However, MA potentiated the increase in the ER stress markers induced by PA after 12 hr of incubation (Figure 4A, 4B, 4C), coinciding with the increment of ceramide levels (Supp Figure 1). Furthermore, consistent with the ability of OA to protect against PA-induced cell death (Figure 2B), OA reduced PA-mediated induction of ER stress markers CHOP and sXBP1 (Figure 4A, 4B); in addition OA prevented PA-induced ROS production (Figure 3A). These protective effects of OA can be accounted for by its capacity to prevent ceramide synthesis and the stimulation of TG accumulation (Figure 1B) thus restraining ceramide formation. Overall these findings suggest that OA protected against PA-induced lipoapoptosis by channeling PA for TG synthesis. In addition, confocal microscopy analyses indicated the nuclear localization of CHOP in response to PA and particularly PA plus MA (Figure 4D). To assess whether ER stress contributed to the potentiation of the lipotoxicity of PA by MA, we determined the effect of chemical chaperones, such as tauroursodeoxycholic acid (TUDCA) that prevents ER stress [27]. As seen, TUDCA reduced the induction of ER stress markers *CHOP* and sXBP1 caused by PA with or without MA (Figure 4E), and protected PMH against PA plus MA-induced lipotoxicity (Figure 4F). In addition, TUDCA treatment reduced ROS generation caused by PA with or without MA (Supplemental Figure 5). However, TUDCA pretreatment did not abrogate the increase in ceramide levels caused by PA with or without MA (not shown). In addition, to examine if *de novo* ceramide generation participates in the ER stress caused by PA and MA, we determined the effect of SPT inhibition by myriocin. We used myriocin to block de novo ceramide synthesis instead of genetic inactivation of CerS to avoid compensatory ceramide generation, as described recently in CerS2^+/−^ and CerS2^−/−^ mice [28, 29]. In line with these data, we observed that CerS2^+/−^ PMH treated with PA with or without MA exhibited enhanced ceramide and glucosylceramide levels and ER stress compared to CerS2^+/+^ PMH (not shown). However, myriocin pretreatment prevented the increase in total ceramide production by PA with or without MA (Figure 5A), and this outcome resulted in the amelioration of ER stress markers CHOP and sXBP1 (Figure 5B, 5C). Thus, these findings suggest a link between *de novo* ceramide generation and ER stress caused by PA and MA.

### Ruthenium red protects against PA and MA-induced lipoapoptosis

To further explore the mechanism of mitochondrial recruitment in the lipoapoptosis induced by PA and MA, we tested the effect of ruthenium red (RR). RR is a known inhibitor of the mitochondrial Ca^2+^ uniporter [30], which imports Ca^2+^ into the mitochondrial matrix to impact mitochondrial function, including mitochondrial permeability transition. In line with previous results in hepatocytes [7], RR protected PMH from PA and PA plus MA-mediated cell death (Supplementary Figure 4C). Overall and consistent with previous findings [7], the data of RR along with the ability of TUDCA to prevent PA-induced ROS suggest a cross-talk between ER and mitochondria in the lipoapoptosis induced by PA, likely *via* Ca^2+^ uptake into mitochondria by a RR sensitive mechanism.

### JNK inhibition protect against PA plus MA-induced cell death

As JNK activation has been shown to play a critical role in PA-mediated lipotoxicity, we next checked the efficacy of JNK inhibition by SP600125. As shown, SP600125 protected primary hepatocytes against PA with or without MA-mediated cell death as well as the expected JNK activation (Supplementary Figure 6). Interestingly, the effect of SP600125 was not potentiated by BHA and TUDCA (not shown), suggesting that JNK activation, ROS generation and ER stress converge in a common pathway.

### Chronic consumption of PA plus MA-enriched diet results in lipodystrophy and progressive liver disease

**Figure 6:**
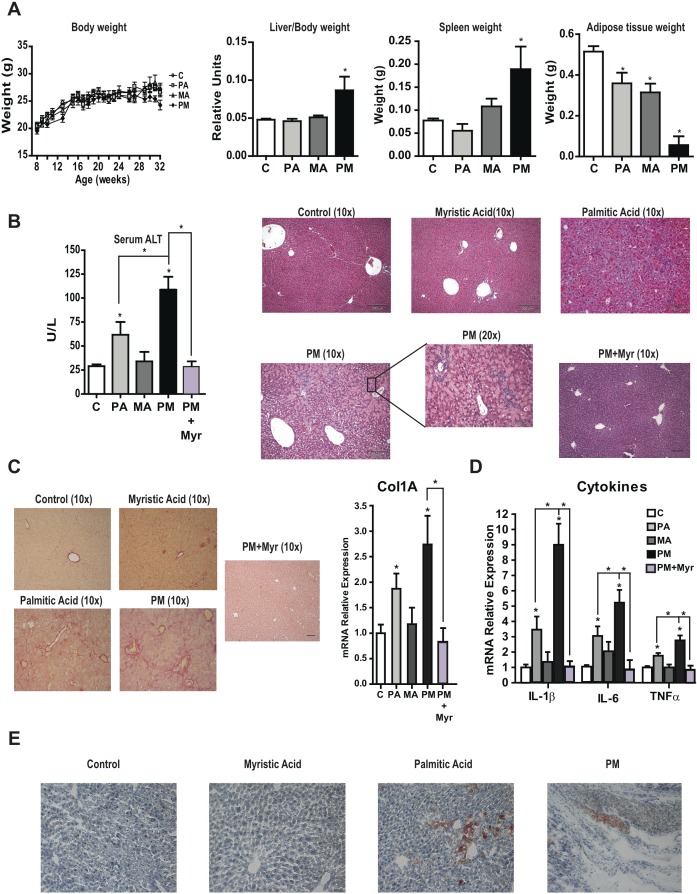
Chronic consumption of diets enriched in PA plus MA leads to lipodystrophy and progressive liver disease and effect of myriocin therapy Mice were fed diets enriched in PA, MA or PA plus MA (PM) for 6 months with or without myriocin treatment in the last 4 months (PM+Myr). Effect on body weigh, liver/body weight, spleen weight and adipose tissue weight were recorded at the end of the study **A.** Serum transaminases levels were determined and liver processed for H&E analyses **B.** at the end of 6 months. Liver sections were staining for Sirius red to determine collagen deposition and expression of Col1α1 was determined by RT-PCR **C.** Inflammatory cytokine expression IL-1β, IL-6 and TNFα was examined in liver samples from mice fed PA, MA or PA plus MA (PM). Liver sections from mice were processed for oil-red staining **E.** Results are the mean±SEM of *N* = 8-10 individual mice. *p < 0.05 *vs*. control and PA-treated PMH.

To address the relevance of the findings in PMH, mice were fed custom-made diets in which fat was derived from PA, MA or their combination (PM) for 6 months. In all cases, required amount of essential fatty acids was ensured by supplementing diets with C18:3 n-3 [19]. A singularity of these custom-made diets compared to high fat diets typically used in obesity studies is that mice did not become obese in any condition (Figure 6A) and exhibited reduced epididymal adipose tissue that was especially pronounced with the diet enriched in PA plus MA, indicating the induction of nutritional lipodystrophy. Moreover, mice fed the combination PA plus MA diet displayed hepatosplenomegaly (Figure 6A). Blood serum analyses showed increased AST and ALT levels in mice fed PA diet that was enhanced by the combination PA plus MA diet indicating increased liver damage (Figure 6B). H&E analyses showed normal appearance in mice fed the diet enriched in MA compared to mice fed diet enriched in PA, which exhibited parenchymal architecture disorganization; these effects were exacerbated in mice fed PA plus MA and were accompanied by inflammatory cell infiltration (Figure 6B) and myeloperoxidase staining. Chronic feeding the PA-enriched diet caused liver fibrosis as indicated by Sirius red staining and increased Col1A1 mRNA levels (Figure 6C), and these effects were magnified in mice fed the combination diet enriched in PA and MA. Moreover, mice fed the PA and MA-enriched diet showed increased expression of inflammatory cytokines IL-1β, IL-6 and TNF compared to mice fed diet enriched in PA (Figure 6D). These findings suggest that the consumption of diets enriched in PA and MA cause lipodystrophy, liver injury, inflammation and fibrosis.

### Mice fed PA plus MA-enriched diet exhibit increased liver ceramide content and ER stress

**Figure 7:**
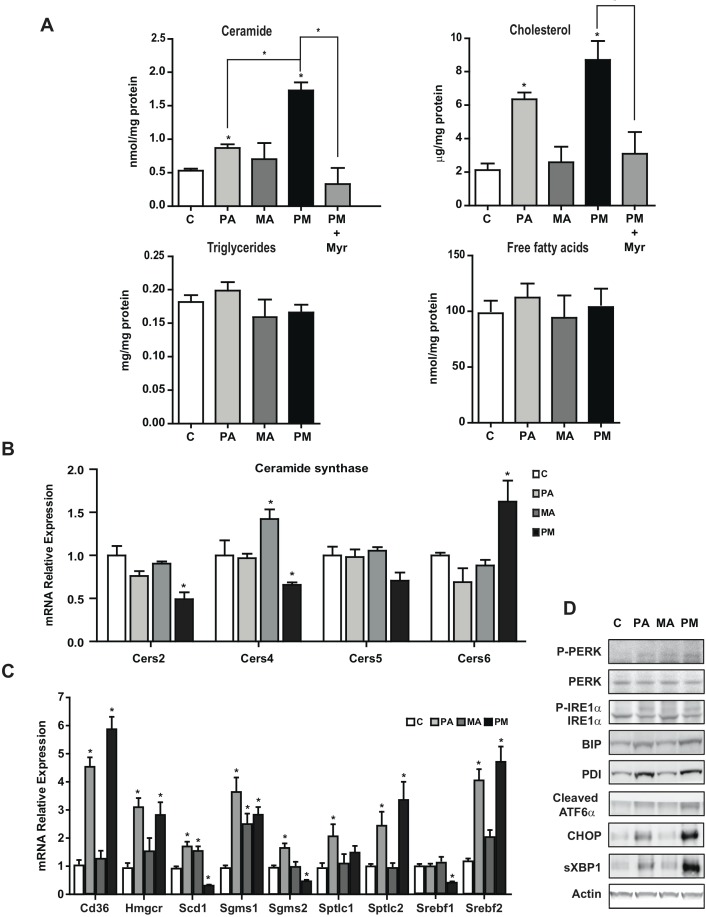
Feeding diets enriched in PA with or without MA increased hepatic ceramide and cholesterol levels and induce ER stress Mice were fed diet enriched in PA with or without MA for 6 months as in Figure 6 and liver samples were processed for hepatic lipid content, ceramide, cholesterol, TG and FFA **A.** In some cases, mice were treated with myriocin (Myr) *in vivo* (i.p. 25mg/kg daily) for the last 4 months of feeding. Expression of hepatic CerS mRNA from mice fed PA, MA or PA plus MA (PM) **B.** Gene expression of enzymes involved in lipid metabolism and ceramide synthesis **C.** Western blot of ER stress markers from mice fed PA, MA or PA plus MA **D.** Results are the mean±SEM of *N* = 8-10 individual mice. *p < 0.05 *vs*. control and PA-treated PMH.

We next analyzed the liver lipid content following the consumption of PA plus MA-enriched diet. Mice fed diets enriched in PA with or without MA showed significant increase in liver cholesterol levels (Figure 7A), without change in the TG content, in line with the mild hepatic steatosis analyzed by Oil-red staining (Figure 6E). Moreover, the hepatic content of FFA did not increase in mice fed the diets enriched in PA, MA or their combination (Figure 7A). However, total hepatic ceramide levels significantly increase in mice fed PA-enriched diet and this effect was exacerbated by the presence of PA plus MA (Figure 7A). This outcome was accompanied by increased expression of CerS6 by PA plus MA compared to diet enriched in PA alone (Figure 7B) and this specific CerS form exhibits preference for 14:0 and 16:0 fatty acids. In addition, PA plus MA decreased the expression of CerS2 and CerS4, which are responsible for the synthesis of C22-C24 and C20 ceramide species, respectively. Gene expression analyses of enzymes involved in lipid metabolism indicated increased expression of the catalytic subunit of SPT, *Sptlc2*, and sphingomyelin synthase *Sgms1* by diets enriched in PA with or without MA (Figure 7C). In addition, *Hmgcr* and *Srebf2* involved in cholesterol synthesis increased in PA and PA plus MA-fed mice (Figure 7C). Moreover, fatty acid transporter *Cd36* expression increased significantly in mice fed PA and PA plus MA diet (Figure 7C). Interestingly, mice fed PA plus MA-enriched diet exhibited reduced expression of *Scd1*, and *Srebf1*, which are involved in FFA synthesis and esterification. These data indicate that hepatic fatty acids are preferentially channeled for ceramide synthesis rather than TG formation. Consistent with *in vitro* findings, PA-enriched diet induced a marked elevation in ER stress markers, which further increased by the combination of PA plus MA (Figure 7D).

In line with findings in PMH, treatment of mice with myriocin prevented PA plus MA-induced ceramide generation and cholesterol content (Figure 7A), resulting in lower liver damage determined by decreased serum ALT levels and H&E analyses (Figure 6B), as well as attenuation of fibrosis reflected by Sirius Red staining and decreased Col1A1 mRNA levels (Figure 6C) and decreased expression of inflammation markers (Figure 6D). Overall, these findings further support the contribution of de novo ceramide synthesis in progressive liver disease caused by feeding diets enriched in PA plus MA.

## DISCUSSION

PA is one of the most abundant saturated fatty acids present in western diets, which is known to cause lipotoxicity by multifaceted mechanisms, including mitochondrial dysfunction, lysosomal destabilization and ER stress [4–7]. Here we describe for the first time the potentiation of PA-induced lipoapoptosis by MA, another SFA, which unlike PA is not lipotoxic. As MA is highly enriched in milk fat and, particularly, in copra/palmist oils, we exposed hepatocytes to a concentration range of MA mimicking that found in these foods and derivatives. While the onset of ER stress and the recruitment of mitochondrial apoptotic pathway induced by PA are potentiated by MA, in line with previous observations with PA alone [6, 7], we focused on the contribution of ceramide synthesis as this particular aspect has not been examined in the presence of PA and MA. The role of ceramide in PA-induced ER stress and lipotoxicity remains poorly characterized has been examined mainly in liver cancer cells and CHO cells [31, 32].

PA fuels *de novo* ceramide synthesis by generating the d18:1 carbon backbone sphinganine from the reduction of 3-ketosphinganine, which is formed by the condensation of palmitoyl-CoA with serine in a reaction catalyzed by SPT; the subsequent acylation of sphinganine with fatty acyl chains of varying lengths generates different ceramide species. Hence, PA not only supports d18:1 sphinganine formation but is the substrate of CerS6, which catalyzes the acylation of sphinganine with PA to generate ceramide 16:0 [13, 14]. In the presence of PA plus MA, two major changes in ceramide homeostasis are observed. First, MA promotes ceramide synthesis from PA and kinetically sustains higher total ceramide levels. In addition, MA competes with PA for the acylation of d18:1 sphinganine, resulting in decreased ceramide 16:0 and higher ceramide 14:0 level, leading to equimolar ceramide14:0 and ceramide 16:0 generation. In line with the reported activation of DES upon its myristoylation at the N-terminus [21], we observed increased stimulation of DES activity using NBD-dehydroceramide 12:0 as substrate, accounting for the sustained increase in total ceramide levels over time. The relative modest generation of ceramide 14:0 by MA alone could be due to the limitation of endogenous PA levels to support d18:1 sphinganine generation to be acylated with MA. Although MA can also act as a substrate for SPT to generate d16:0 sphingolipids, this process is limited by the lower affinity of the SPT complex for MA compared to that of PA [12, 18, 32, 33]. The expression of the catalytic subunit STPLC3 broadens the substrate specificity of SPT allowing utilization of myristoyl-CoA for synthesis of d16:0 carbon backbone [34]. However, expression of SPTLC3 subunit is barely detectable in normal liver, although its levels increase in hepatocellular carcinoma [35].

The switch in ceramide profile by PA *vs* PA plus MA resulting in increased ceramide 14:0 and decreased ceramide 16:0 is of relevance in the lipotoxicity of PMH. Interestingly, PMH are more sensitive to equimolar ceramide 14:0 and ceramide 16:0, mimicking the profile elicited by PA plus MA, than to either ceramide species alone. This increased susceptibility may reflect an emerging prosurvival role of ceramide 16:0, which has been recently described in cancer cells by regulating ER stress response [36]. While MA is not hepatotoxic by itself, it has been recently reported that myristate is cardiotoxic and induced cardiomyocyte hypertrophy in an autophagy-dependent manner [22]. Further investigation will be required to decipher the tissue specific cytotoxic potential of MA and whether it is related to differential ceramide species generation.

**Figure 8:**
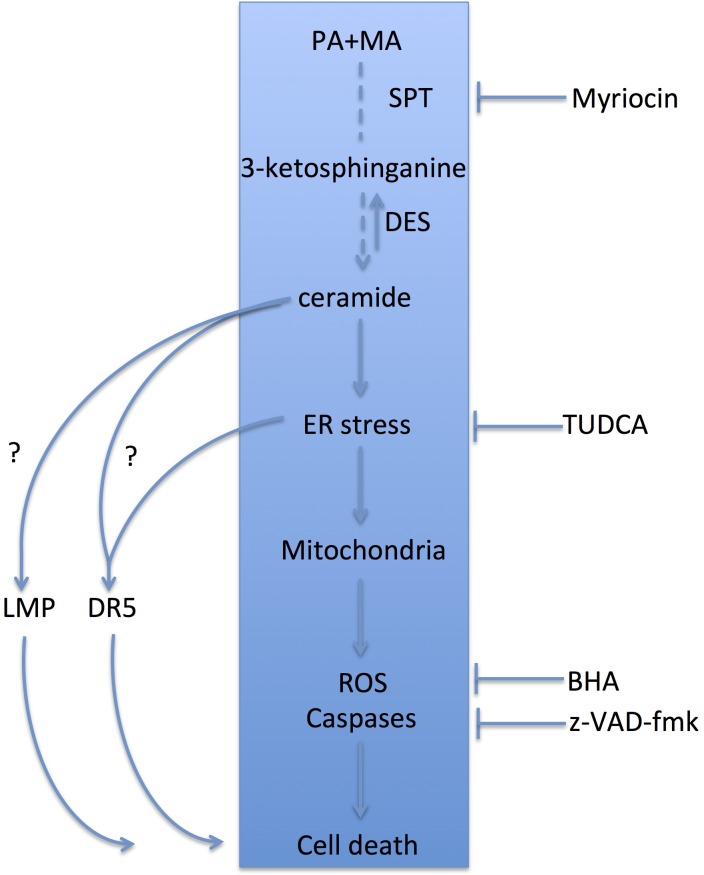
Schematic proposal of the molecular events involved in the lipotoxic effects of PA and the potentiation by MA PA fuels ceramide synthesis by providing sphinganine in a reaction catalyzed by SPT. MA in turn potentiates ceramide generation by stimulating the activity of DES, and therefore the combination of MA plus PA results in kinetically sustained de novo ceramide production. This event is upstream of the induction of ER stress, which in turn recruits the mitochondrial apoptosis program eliciting ROS generation and caspase activation, leading to hepatocyte apoptosis. On the hand right side, we show the different specific inhibitors used to interrupt this signaling cascade. In addition to ceramide generation, PA is also known to activate other cellular events, including lysosomal membrane permeabilization (LMP) and activation of death receptor 5 (DR5), which then contribute to PA-induced lipoapoptosis. While myriocin blocks the central events shown (blue rectangle) it is not expected to interfere with LMP and DR5 activation. Further work is required to examine the involvement of ceramide synthesis in the recruitment and activation of LMP and DR5 by saturated fatty acids.

The potentiation of PA-induced lipoapoptosis by MA involves ER stress and mitochondrial recruitment by a mechanism dependent of mitochondrial Ca^2+^ import sensitive to RR. Moreover, antioxidant BHA and caspase blockade protect against PA plus MA-mediated cell death. Inhibition of ER stress by TUDCA protects against PA and MA-induced cell death but does not abrogate ceramide synthesis. Furthermore myriocin, a specific SPT inhibitor, protects against PA plus MA-induced ceramide generation and subsequent ER stress induction and hepatocyte apoptosis. Overall, these findings, indicate that PA plus MA-induced ceramide generation is upstream of ER stress and mitochondrial recruitment. While these findings suggest a *de novo* ceramide synthesis-ER stress-mitochondria axis contributing to PA-induced lipoapoptosis with potentiation by MA, protection with myriocin or TUCDA is partial, indicating the participation of other pathways independent of *de novo* ceramide synthesis and ER stress. In this regard, PA has been shown to elicit hepatocyte apoptosis through lysosomal membrane permeabilization [3, 37] or DR5 activation [8,9], which could account for the partial protection afforded by myriocin. In addition, a direct effect of ceramide in mitochondria causing ROS and apoptosis has been reported in different cell tpes [13, 38–40], which may underlie the residual cell death in the presence of TUDCA. Further work will be required to examine the contribution of ceramide in PA-induced lysosomal membrane permeabilization and DR5 activation (Figure 8).

The role of ceramide in PA-induced lipotoxicity and ER stress is controversial. For instance, Listenberger *et al.* claimed that PA-induced cell death in CHO cells did not depend on ceramide synthesis [31]. However, the inhibition of SPT and CerS inhibition with L-cycloserine and fumonisin B1, respectively, decreased caspase-3 activity and reduced DNA degradation. Moreover, Wei *et al.* discarded the contribution of *de novo* synthesized ceramide in PA-induced lipotoxicity in the H4IIE hepatic cell line treated with fumonisin B1 [32]. As expected, fumonisin B1 resulted in increased dihydrophingosine generation by PA, which has been shown to mediate the toxicity of fumonisin B1 in different cell types [41, 42]. In addition to these possibilities, the difference between tumor cell lines and primary hepatocytes could also contribute to the final outcome.

To explore the relevance of the findings in PMH, we fed mice diets enriched in PA with or without MA. In line with the *in vitro* observations, the chronic consumption of these diets, mimicking the ingestion of copra/palmist oils and western foods, resulted in increased liver ceramide content and ER stress, effects that were exacerbated by the combination PA plus MA, and this outcome was accompanied by lipodystrophy, enhanced liver injury, inflammation and fibrosis. Consistent with the findings in PMH, there was negligible macrovesicular steatosis indicating that dietary PA and MA were diverted from TG synthesis and preferentially converted in ceramide species. This outcome mirrored the increased expression of the catalytic subunit of the SPT complex SPTLC2 and CerS6, which exhibit preferential affinity for MA and PA, as well as the downregulation of SREBP-1 and SCD-1. Besides ceramide accumulation, PA plus MA diet also increased liver cholesterol levels, which may be consequence of ceramide-induced ER stress and subsequent activation of the transcription factor SREBP-2 and the enzyme catalyzing the rate-limited step in cholesterol synthesis, HMGCoAR. Thus, despite absence of macrosteatosis and TG deposition, nutritional intake of PA plus MA resulted in the selective accumulation of ceramide and cholesterol that may account for the observed hepatomegaly. Another key feature of this feeding regimen was the marked loss of adipose tissue, underlying the nutritional lipodystrophy induction by PA plus MA, which contrasts with the outcome described with high fat diets typically used for obesity and glucose homeostasis studies, suggesting that the nutritional fat was diverted to the liver for conversion into ceramide. Interestingly, our findings are similar to those reported recently in mice fed an atherogenic diet enriched in cholesterol (1.25%) with the loss of adipose tissue, accumulation of liver cholesterol, inflammation, fibrosis and liver injury [43].

These *in vivo* findings may have important implications. While most studies investigating the effects of SFA have focused in coronary heart disease and atherosclerosis [44], our results imply that people consuming diets enriched in PA and MA may develop lipodystrophy and chronic liver disease, characterized by the lack of TG accumulation and macrovesicular steatosis and the preferential accumulation of ceramide and cholesterol levels, contributing to hepatomegaly and liver injury. Moreover, unlike other high fat diets consumption of diets enriched in PA plus MA does not result in increased body weight although it induces inflammation and fibrosis indicative of progressive NAFLD. Interestingly, recent studies have reported that children of Asian origin exhibit similar abnormal liver function tests but increased prevalence of moderate to severe fibrosis compared to Caucasians despite decreased body mass index [45]. Further investigation is needed to ascertain whether differential nutritional intake and consumption of PA plus MA in this children population can account for this outcome.

## MATERIALS AND METHODS

### Mice, hepatocyte isolation and treatments

Wild type mice (C57BL/6J, Charles River Laboratories, Wilmington, MA) and CerS2^+/−^ mice (obtained from Drs. A. Futerman and S. Summers) were used as the source of PMH and were isolated as previously described [46]. PMH were treated with PA, MA or OA alone or in combination at equimolar concentrations (0.25-1mM) for 1-12 hours. Stock solutions of PA, MA or OA were prepared as described [6] and the final working concentrations were made with 0.5-1% (w/v) fatty acid free bovine serum albumin (BSA, Roche). FFA:BSA ratio ranged from 3:1 to 6:1 depending on the final FFA concentration. High FFA:BSA ratio has been observed in states of insulin resistance and obesity, two risk factors for NASH [47]. In some cases, PMH were treated with butylated hydroxyanisole (BHA, 50 - 200 μM) (Sigma-Aldrich) in absolute ethanol, z-VAD-FMK (5 - 15 μM) (Abcam, Cambridge, UK) in dimethyl sulfoxide (DMSO), myriocin (20 μM) (Enzo Life Sciences, Farmingdale, NY) in NaOH (50 mM) and DMSO (0.1%), tauroursodeoxycholic acid (TUDCA, 25 μM) (Calbiochem, Billerica, MA), ruthenium red (RR, 10 μM) and the JNK inhibitor SP600125 (20μΜ). Cell death, lipodomic analyses, total ceramide levels, DES activity from NBD-dihydroceramide C12; ROS generation and determination of ER stress are described in Supplemental Material section.

### Ceramide analysis by HPLC

Cellular ceramide levels were determined by high performance liquid chromatography (HPLC) after derivatization of the sphingoid base with O-phthaldehyde following deacylation of ceramide, as described previously [46]. Briefly, cells were collected in PBS and lipids were extracted with methanol:chloroform (1:2, v/v) isolation. The organic phase was resuspended in 250 μL of 1 N KOH in methanol, and incubated 1 hour at 100°C, yielding free sphingoid base, followed by derivatization with O-phthaldehyde. Samples were centrifuged and analyzed by HPLC in a reverse phase C18 column (Teknokroma) using a Gilson fluorimetric detector (Middleton, WI) with an excitation wavelength of 252 nm and an emission wavelength of 483 nm. The mobile phase for the gradient system was 5 mM potassium phosphate buffer (pH = 6.5):methanol (85:15, v/v) for mobile phase A and acetonitrile:methanol (75:25, v/v) for mobile phase B and the flow rate was 1 mL/min. The gradient program was 0-1 min 52.94 A, 47.06 B; 1-6 min 52.94-5.88 A, 47.06-94.11 B; 6-21min 5.88 A, 94.11 B; 21-25 min 5.88-52.94 A, 94.11-47.06 B, 25-30 min 52.94 A, 47.06 B. Quantification of ceramide peak was calculated according to a calibration curve derived from commercial purified standards and values were normalized by protein quantification.

### Ceramide species analysis with mass spectrometry

Lipids from homogenate samples were extracted with methanol:chloroform isolation (1:2, v/v). Mass spectrometry analysis of lipid species was performed in the Research Unit on Bioactive Molecules at the Institute of Advanced Chemistry of Catalonia (IQAC). Cells or tissue homogenates were pelleted, washed in PBS, and transferred to glass vials. Sphingolipid extracts were spiked with internal standards (N-dodecanoylsphingosine, N-dodecanoylglucosylsphingosine, and N-dodecanoylsphingosylphosphorylcholine, 0.2nmol each) and analysed in a Waters Aquity UPLC system connected to a Waters LCT Premier orthogonal accelerated time of flight mass spectrometer (Waters, Millford, MA) operated in positive electrospray ionisation mode. The analytical column was a 100mm x 2.1mm i.d., 1.7μm C8 Acquity UPLC BEH (Waters). The two mobile phases were phase A: water/formic acid (500/1 v/v); phase B: methanol/formic acid (500/1 v/v), both also contained 5mM ammonium formate. A linear gradient was programmed— 0.0min: 80% B; 3min: 90% B; 6min: 90% B; 15min: 99% B; 18min: 99% B; 20min: 80% B. The flow rate was 0.3mL/min. The column was maintained at 30°C. Quantification was carried out using the extracted ion chromatogram of each compound, using 50mDa windows. The linear dynamic range was determined by injecting standard mixtures.

### Determination of dihydroceramide desaturase and serine palmitoyltransferase activities

The determination of DES activity was performed as described in detail previously [21]. Briefly, cell extracts were incubated with NBD-dihydroceramide C12:0 and the resulting synthesized ceramide 12:0 was resolved from the precursor dihydroceramide by HPTLC as described previously [21]. SPT activity was determined as described previously [24], based on the conversion of water-soluble [3H]serine to the chloroform-soluble product, 3-ketodihydrosphingosine.

### *In vivo* experiments

Studies were conducted in accordance with the principles and procedures outlined in the National Institutes of Health (NIH) Guide for the Care and Use of Laboratory Animals and were approved by the institutional animal care committee of the Universitat de Barcelona. Eight weeks old male mice (C57BL/6J, Charles River Laboratories) were fed custom-made fat diets *ad libitum* (Research Diets, New Brunswick, NJ), enriched in PA (30% of Kcal), MA (30% of Kcal) or their combination (PM) as the lipid source, complemented with the presence of essential the fatty acid C18:3 n-3 [19] for 6 months. In some cases, mice fed the PA plus MA enriched diet were treated with myriocin (i.p. 25mg/kg dayly) for the last 4 months of feeding to examine the impact of blocking ceramide synthesis in liver disease.

### Statistical analysis

Results are expressed as mean ± standard error of the mean (SEM). Statistical significance of mean values has been assessed using Student T-test and one or two-way ANOVA. Statistics were performed using GraphPad Prism 6 software.

## SUPPLEMENTARY MATERIAL FIGURES



## Abbreviations

NASH: non-Alcoholic steatohepatitis
ER: endoplasmic reticulum
SPT: serine palmitoyltransferase
PA: palmitic acid
CERS: (dihydro)ceramide synthase
DES: dihydroceramide desaturase
MA: myristic acid
GCS: glucosylceramide synthase
NAFLD: non-alcoholic fatty liver disease
FFA: free fatty acid
OA: oleic acid
BSA: bovine serum albumin
C: control
S: solvent
BHA: butylated hydroxyanisole
DMSO: dimethyl sulfoxide
TUDCA: tauroursodeoxycholic acid
PBS: phosphate buffered saline
ATF6α: activating transcription factor alpha
CHOP: CCAAT-enhancer-binding protein homologous protein
HPLC: high performance liquid chromatography
NDA: naphthalene-2 3-dicarboxaldehyde
TG: triglyceride
GST: glutathione-S-transferase
CDNB: 1-cloro-2 4-dinitrobenzene
DCF: dichlorofluorescein
SEM: standard error of the mean
PMH: primary mouse hepatocytes
SFA: saturated fatty acid
UPR: unfolded protein response
TNFα: tumor necrosis factor alpha
IL: interleukin
