# Supplementary material for: Myristic acid potentiates palmitic acid-induced lipotoxicity and steatohepatitis associated with lipodystrophy by sustaning de novo ceramide synthesis
Source: Oncotarget. 2015 Nov 2;6(39):41479–96. doi: 10.18632/oncotarget.6286 (PMC4747168; doi:10.18632/oncotarget.6286)
Supplement: Supplementary file 1 [file oncotarget-06-41479-s001.pdf]

Myristic acid potentiates palmitic acid-induced lipotoxicity and steatohepatitis associated with lipodystrophy by sustaning de novo ceramide synthesis

Supplementary Material

SUPPLEMENTAL METHODS

Protein analysis. After treatments cells were washed with phosphate buffered saline (PBS) (Gibco, Waltham, MA) and scrapped with RIPA commercial buffer (Sigma-Aldrich) supplemented with inhibitors of proteases and phosphatases. Proteins were quantified with the Bradford spectrophotometric assay (Bio-Rad, Hercules, CA) and 40 µg of each sample was used for immunoblot analysis. The following antibodies have been used: anti-βActin-HRP (Sigma-Aldrich), anti-ATF6α (Acris, Herford, Germany), anti-CHOP, anti-cleaved caspase 3, anti-Cleaved PARP, anti-PDI, anti-PERK and anti-Phospho-PERK^{Thr980} were from Cell Signaling Technology (Danvers, MA), anti-GRP78 (Stressgen ADI-SPA-826), anti-XBP1 (Santa Cruz Biotechnology, Santa Cruz, CA), anti-Rabbit-HRP (Sigma-Aldrich), anti-Mouse-HRP (Sigma-Aldrich).

Triglycerides quantification and Oil Red staining. Intracellular lipid accumulation was determined by oil red staining in cells fixed with paraformaldehyde. Briefly, cells were stained for 15 minutes with oil red working solution diluted with distilled water. Excess oil red was extracted by 30 seconds exposure to isopropanol 60% and cells were counterstained with hematoxylin for 4 minutes following a 5 minutes exposure to acetic acid 4%. Stained cells were embedded in Aquatex® (Merck, Whitehouse Station, NJ) and observed in the optic microscope.

Triglycerides (TG) from cell lysates were analyzed in the Clinical Core Laboratories at the *Hospital Clínic i Provincial de Barcelona* on an ADVIA 2400 Chemistry System (Siemens Medical Solutions, Erlangen, Germany) using commercial available kits,

ADVIA Chemistry System 1650 (Bayer-Siemens) and CHOD-PAP (Roche Diagnostics). TGs levels were corrected according to protein content determined by Bradford colorimetric assay.

Quantitative Reverse Transcription PCR. RNA was isolated using Trizol reagent (Invitrogen, Waltham, MA). Quantitative PCR analysis was performed using SYBR Green (Bio-Rad) in a real-time PCR system (iCycler Thermal Cycler, Bio-Rad). The thermal cycling program was 10 min at 50°C for the cDNA formation, 5 min at 95°C for enzyme activation and 40 cycles of denaturation for 10 seconds at 95°C, 35 seconds annealing at 57°C, and 25 seconds extension at 72°C. To normalize expression data, 18S or β-actin were used as internal control genes.

Cellular viability assessment. For trypan blue cell death analysis, cells were trypsinized and carefully resuspended in 0.96% w/v Krebs-Henseleit (Sigma-Aldrich). Cells were diluted 3 times with 0.2% trypan blue and counted in a Newbauer chamber under the optical microscope. Percentage of viability was obtained from dividing the number of live cells (unstained) by the number of total cells (unstained plus blue stained). Alternatively, cultured cells were incubated with propidium iodide or Hoechst 0.001% w/v and observed under a fluorescent microscope with an excitation wavelength of 530 nm and 504 nm respectively. Propidium iodide penetrates only in death cells while Hoechst is able to enter also live cells and stains DNA.

Cell viability was also assessed by glutathione-S-transferase (GST) activity spectrophotometric assay. Briefly, medium fraction was collected and cells were scrapped with 10% triton X-100 in PBS. Samples were vortexed and centrifuged for 5 minutes at 12000 rpm and measured in the spectrophotometer at 340 nm. The activity assay buffer was 75% of miliQ water, 20% of 0.5M Na_2HPO_4, pH = 6.5 and 5% of 1.97 mM 1-cloro-2,4-dinitrobenzene (CDNB) in absolute ethanol. The assay was performed

in a 4 mL cuvette with 100 µL of sample, 3 mL of buffer with CDNB and 100 µL of GSH 40 mM as enzyme cofactor. A blank without sample was done and absorbance was measured every 2 seconds during 40 seconds. Cell viability percentage was obtained by dividing the slope of the cell extract by the sum of cell extract plus medium fraction.

Cytochrome c release assessment. PMHs were cultured at a density of $1.5x10^6$ cells in 60 mm dishes and incubated with 1 mM FFAs for 12 hours. Afterwards, cells were trypsinized for 1 min and remained without trypsin for 2 min. Then hepatocytes were carefully collected in PBS at 4°C and pelleted by 1 min centrifugation at 12000 rpm. Intact cells were carefully resuspended with 125 µL of PBS and incubated for 1 min in a 25 µL drop of 0.15% (w/v) digitonin dissolved in manitol buffer (250 mM manitol, 19.8 mM EDTA, 17 mM hepes, adjusted to pH = 7.5) at 37°C that was placed in a microtube with silicon:paraffin (6:1). Tubes were immediately centrifuged for 1 min at 13000 rpm to separate the cytosolic fraction (upper drop) from the mitochondrial fraction pelleted in 25 µL of trichloroacetic acid 10%.

Caspase 3 activity. In some cases, we determined caspase 3 activity by release of 7-amino-4-trifluoromethyl coumarin from Ac-DEVD-AMC and fluorescence was continuously recorded with emission at 460 nm and excitation at 355 nm.

Oxidative stress measurement. Reactive oxygen and nitrogen species were measured by the fluorescence produced when they bind to dichlorofluorescein-diacetate (DCF-DA) (Sigma-Aldrich). DCF-DA (10 µM) was freshly added to the culture media and incubated for 30 minutes at 37°C in the dark. Afterwards, cells were rinsed, collected with PBS and placed in a black 96-well plate to measure fluorescence in a fluorimeter at 495 nm excitation and 525 nm emission wavelengths. The induction of reactive species was calculated with respect to control samples.

Confocal microscopy. Confocal microscopy was used to analyze colocalization of CHOP transcription factor with nuclei of primary hepatocytes. Cells were incubated with Hoechst for 10 minutes and then fixed for 15 minutes with paraformaldehyde 4%. Cells were permeabilized with 0.2% saponin and blocked with 1% BSA fatty acid free for 15 minutes. CHOP antibody 1:500 (Cell Signaling) was incubated overnight in BSA 1% followed by a secondary antibody incubation for 1 hour at room temperature in BSA 0.1%. Stained samples were embedded in fluoromont (Sigma-Aldrich) and digital images were taken in a confocal microscope.

In vivo studies.

Blood was harvested from the lower cava vein with heparinized 18G needles in order to avoid hemolysis, and centrifuged at 4°C for 10 min at 10000 rpms to collect serum from the top phase. Serum was diluted 1/2 with saline and samples were analyzed in the Clinical Core Laboratories at the *Hospital Clínic i Provincial de Barcelona*, to measure transaminases (alanine aminotransferase (ALT) and aspartate aminotransferase (AST)), cholesterol, triglycerides and free fatty acids.

Liver was harvested and cut in transversal slices. A slice of medium, left and right lobe were kept in formalin for 48h and embedded in paraffin in an automatic inclusor for microtome sections. For Hematoxylin&Eosin staining 7 µm sections were made. For protein and lipid analysis, 100 mg of liver were homogenized in 900 µL of Homobuffer (70 mM sucrose, 220 mM manitol, 2 mM Tris-HCl, pH = 7.4, 0.1 mM EDTA, 0.1% BSA fatty acid free). For protein, 25 µL of homogenized tissue was diluted 1/2 with RIPA buffer supplemented with antiproteases and antiphosphatases, and samples were analyzed by immunoblot. For ceramide HPLC quantification, 4 mg of protein of each sample were used for lipid extraction. Homogenates were also diluted ½ with homobuffer and analyzed in the Clinical Core Laboratories at the Hospital Clínic de

Barcelona to measure liver cholesterol, TG and FFA. Finally, a small piece of right lobe was homogenized in Trizol reagent to extract mRNA.

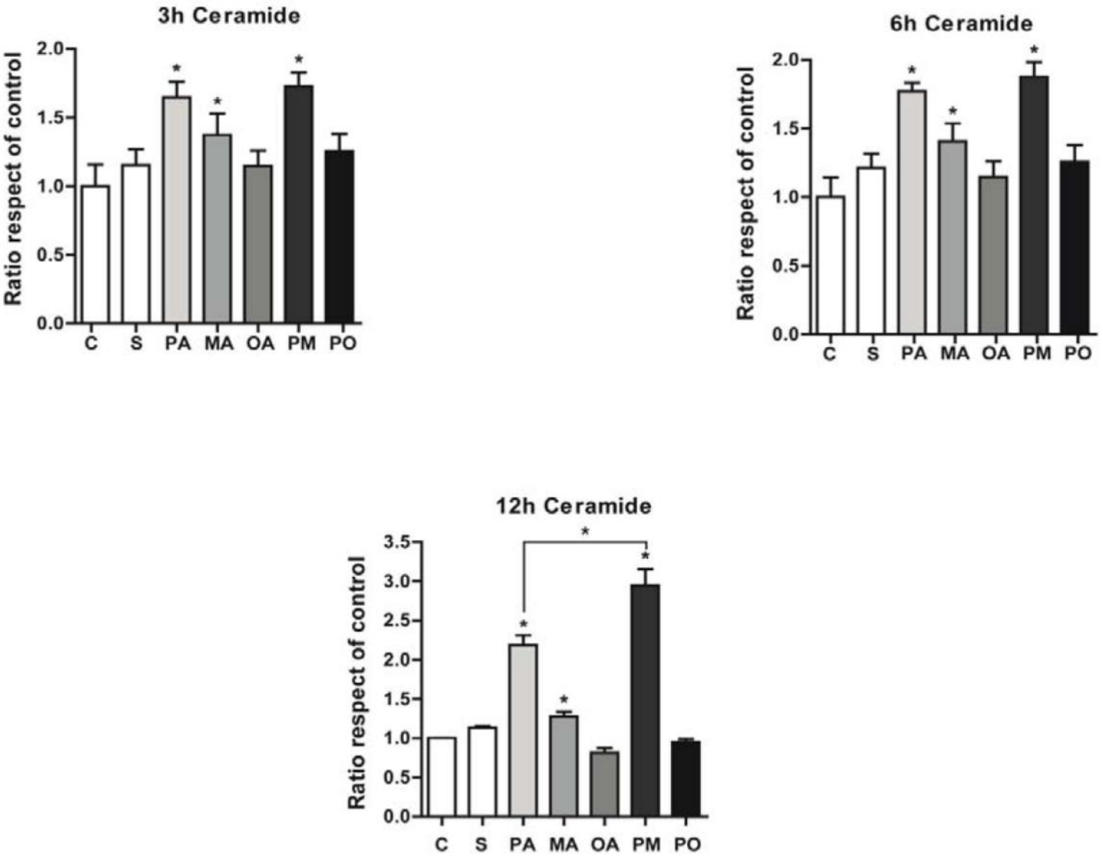

SUPPL FIG 1. Kinetics of total ceramide levels in response to PA, MA and PA plus MA.

PMH were treated with PA, MA or PA plus MA (PM) (0.5mM) each at the indicated periods of time (3-12 hours) and total ceramide levels were determined by HPLC as described in Supplemental methods. Results are the mean±SEM of N=5-7 individual experiments. *$p<0.05$ vs. control and PA-treated PMH.

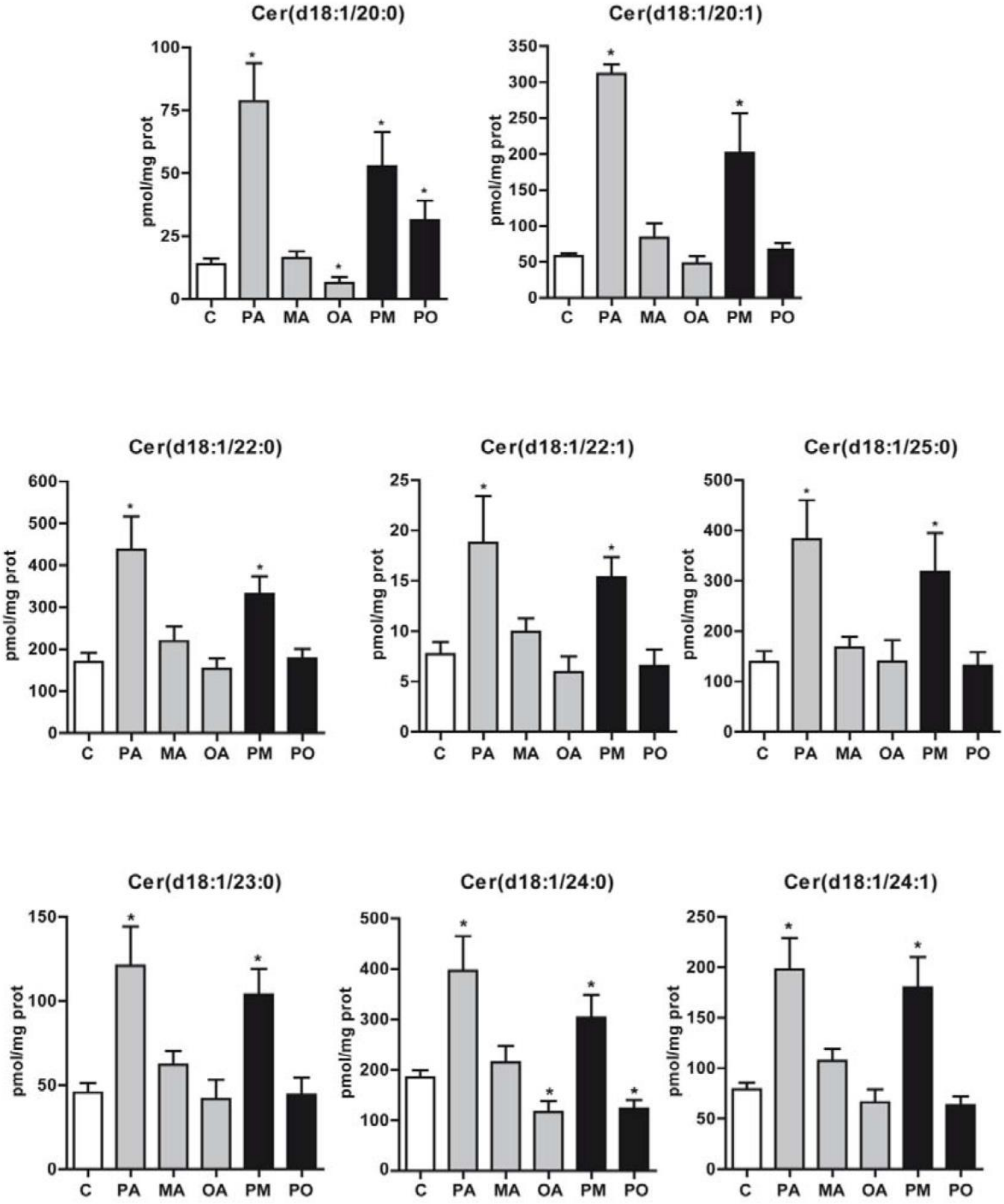

SUPPL FIG 2. Long-chain ceramide species in PMH following incubation with PA, MA or PA plus MA.

PMH were treated with with PA, MA or PA plus MA (PM) (0.5mM) for 12 hours and lipidomic profile was performed by mass spectrometry analyses as described in Supplemental methods. Results are the mean±SEM of N=5-7 individual experiments. *p<0.05 vs. control and PA-treated PMH.

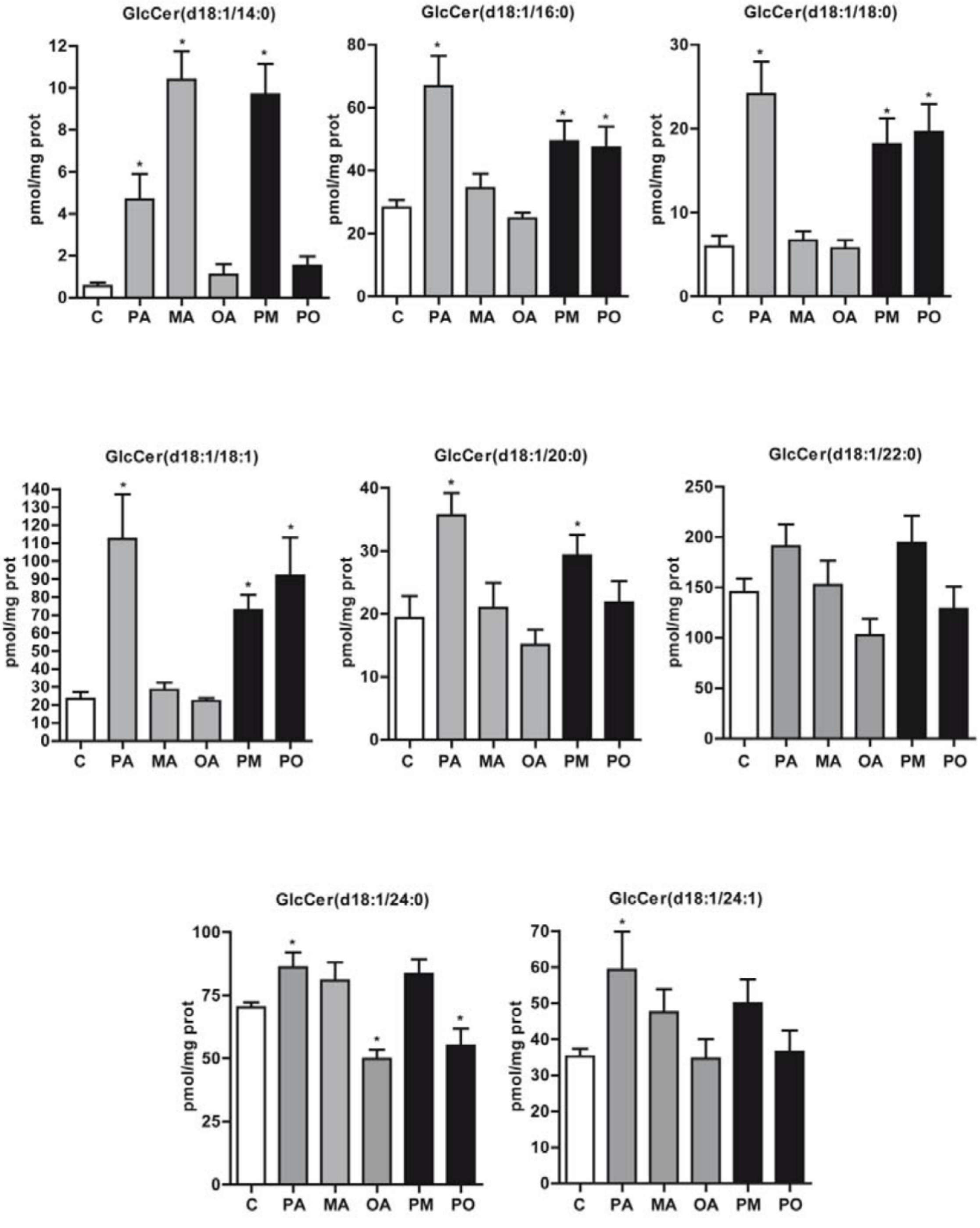

SUPPL FIG 3 Glucosylceramide species in PMH after incubation with PA, MA or PA plus MA.

PMH were treated with with PA, MA or PA plus MA (PM) (0.5mM) for 12 hours and glucosylceramide profile determination was performed by mass spectrometry analyses as described in Supplemental methods. Results are the mean±SEM of N=5-7 individual experiments. *$p<0.05$ vs. control and PA-treated PMH.

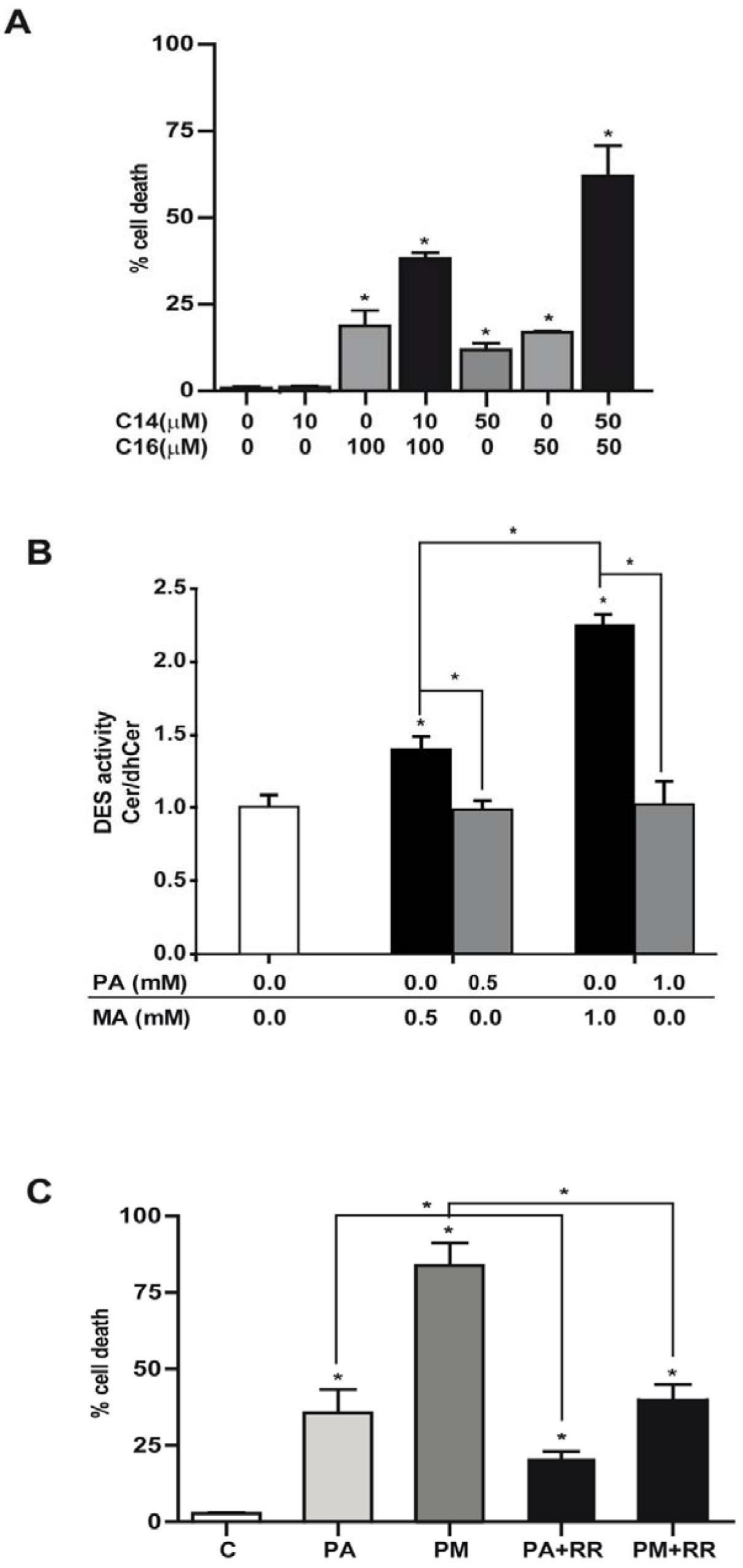
A
100
75
50
25
0
% cell death
C14(μM) 0 10 0 10 50 0 50
C16(μM) 0 0 100 100 0 50 50
B
2.5
2.0
1.5
1.0
0.5
0.0
DES activity
Cer/dhCer
PA (mM) 0.0 0.0 0.5 0.0 1.0
MA (mM) 0.0 0.5 0.0 1.0 0.0
C
100
75
50
25
0
% cell death
C
PA
PM
PA+RR
PM+RR

SUPPL FIG 4. Effect of ceramide 14:0 and ceramide 16:0 on cell death, activation of DES activity by MA and protection by ruthenium red against cell death.

PMH were incubated with exogenous ceramide 14:0, ceramide 16:0 and ceramide 14:0/ceramide 16:0 at the indicated concentrations to examine their impact in cell death (A). Concentrations shown of these ceramide species were based on the mass spectrometry levels determined in Fig 1D following PA, MA or PA plus MA. (B) The stimulating effect of PA or MA in DES activity was assayed in PMH using NBD-dihydroceramide 12:0 as substrate. Resolution of dihydroceramide C12:0 from ceramide C12:0 was determined by HPTLC as described before [21]. (C) Effect of ruthenium red (RR, 10μM) on cell death following PA and PA plus MA (PM). Results are the mean±SEM of N=3-5 individual experiments. *$p<0.05$ vs. control and PA-treated PMH.

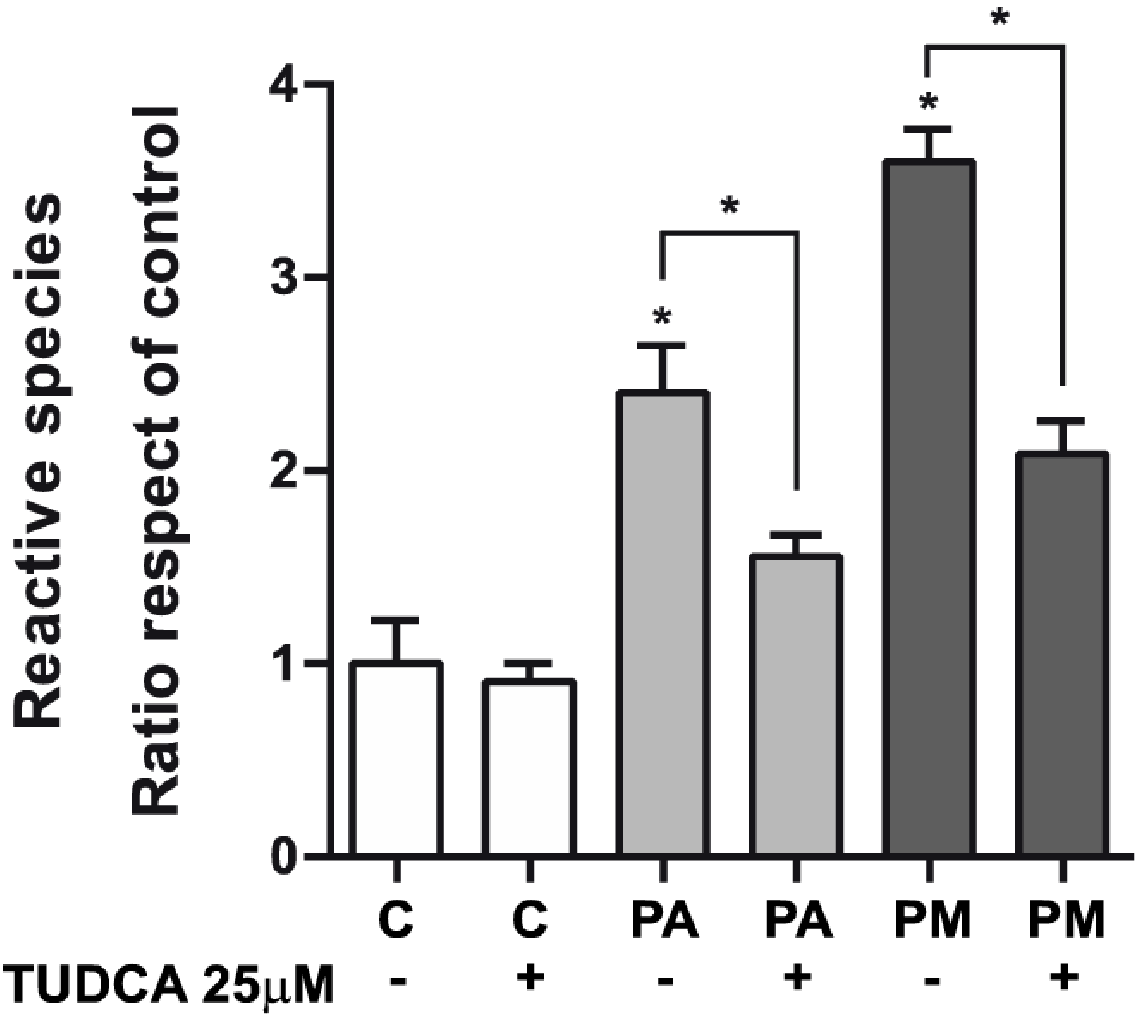

SUPPL FIG 5. Effect of TUDCA on ROS generation in PMH challenged with PA, MA and PA plus MA.

PMH were incubated with with PA, MA or PA plus MA (0.5mM) for 12 hours with or without TUDCA treatment to determine ROS generation by DCF-DA (10 µM). Results are the mean±SEM of N=3 individual experiments.*$p<0.05$ vs. control and PMH in the absence of TUDCA.

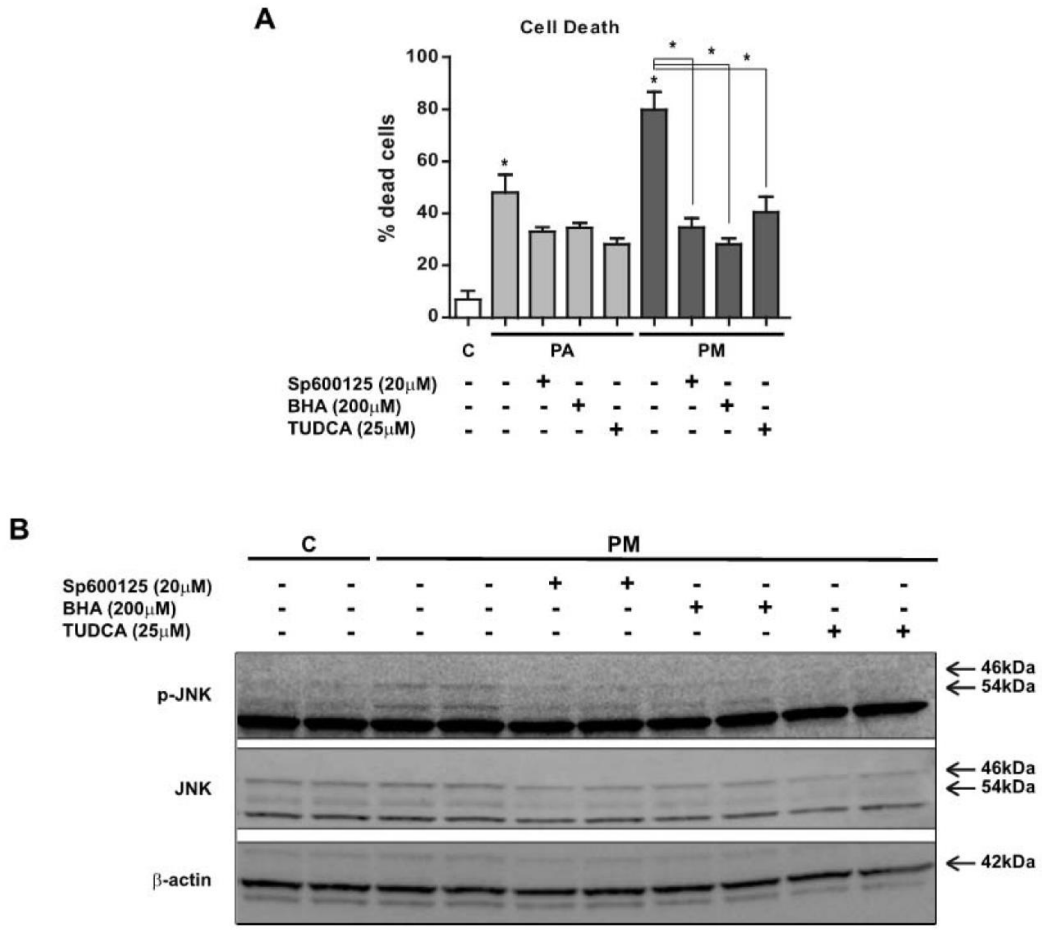

SUPPL FIG 6. Effect of JNK inhibitor SP600125 in PA and PA plus MA mediated cell death.

PMH were incubated with with PA and PA plus MA (0.5mM) for 12 hours with or without SP600125, BHA or TUDCA and cell death was determined by the release of extracellular GST in the medium (A). Cell extract 4 hours after PA or PA plus MA incubation were processed for phosphor-JNK expression (B). Results are the mean±SEM of N=4 individual experiments *p<0.05 vs. control PA or PA plus MA PMH.
